# Medical comorbidities and dementia diagnostic outcomes in African American and Hispanic individuals enrolled in DAWN

**DOI:** 10.1002/alz70860_106956

**Published:** 2025-12-23

**Authors:** Katalina F. McInerney, Andrew F Zaman, Pedro R Mena, Larry D Adams, Jose J. Sanchez, Vanessa C. Rodriguez, Glenies S. Valladares, Katrina Celis, Kara L Hamilton‐Nelson, Farid Rajabli, Michael B. Prough, Gary W Beecham, Gary W Beecham, Gary W Beecham, Briseida E. Feliciano‐Astacio, Jeffery M Vance, Margaret Pericak‐Vance, Michael L Cuccaro

**Affiliations:** ^1^ Department of Neurology, University of Miami Miller School of Medicine, Miami, FL, USA; ^2^ John P. Hussman Institute for Human Genomics, University of Miami Miller School of Medicine, Miami, FL, USA; ^3^ University of Miami Miller School of Medicine, Miami, FL, USA; ^4^ Dr. John T. Macdonald Foundation Department of Human Genetics, University of Miami Miller School of Medicine, Miami, FL, USA; ^5^ Department of Biostatistics and Data Science, Wake Forest School University of Medicine, Winston‐Salem, NC, USA; ^6^ Universidad Central del Caribe, Bayamón, PR, USA

## Abstract

**Background:**

It is well established there is a complex relationship between brain health and medical comorbidities such cardiovascular and metabolic health conditions. However, the relationship between these medical conditions and ADRDs in community dwelling Hispanic (HI) and African American (AA) individuals is not yet well understood. In this study we aim to assess the cognitive impact of cardiovascular and metabolic conditions on HI and AA individuals ascertained in the community.

**Method:**

Our sample of 1908 participants (890 HI, 1018 AA) was predominantly female (80%) with mean educational attainment of 13.1 ± 4.1 years. Participants were classified as non‐cognitively impaired (NCI; *N* = 949) or cognitively affected (*N* = 959; MCI *N* = 734; AD *N* = 225) by consensus panel.

A binary logistic regression was performed to determine what factors increased the risk of being classified as cognitively affected. Our independent predictor variables were group (AA, HI), age, sex (male/female), years of education, number of cardiovascular risk factors, and number of metabolic risk factors.

**Result:**

Our ordinal regression model demonstrated good fit (*X^2^* = 56.75, *p* < 0.001). Individuals with more cardiovascular (*b* = 0.20, Wald=5.13, *p* = 0.02), and metabolic risk factors (b=0.11, Wald=3.99, *p* <0.05), had a higher chance of being classified as cognitively affected. Increased age (b = 0.02, Wald = 7.99, *p* = 0.005), sex (male; b=0.36, Wald=12.92, *p* <0.001) and ethnicity (HI; b = 0.26, Wald = 4.92, *p* = 0.03) were all associated with an increased risk of being classified as cognitively affected. Educational attainment was not statistically significant (b=‐0.02, Wald=3.51, *p* = 0.06), but there was a trend for more years of education to be protective.

**Conclusion:**

Cardiovascular and metabolic conditions significantly increase the risk of classification as cognitively affected in community dwelling HI and AA individuals. Of note, this risk did not differ between our HI and AA groups. These results suggest the need to raise awareness about the impact of medical comorbidities on brain health in HI and AA communities. Such comorbidities represent preventable and reversible risk factors for cognitive decline. An important next step will be to integrate these phenotypes into genetic studies of ADRDs.

